# Standards for Quantitative Measurement of DNA Damage in Mammalian Cells

**DOI:** 10.3390/ijms24065427

**Published:** 2023-03-12

**Authors:** Donald H. Atha, Vytas Reipa

**Affiliations:** Materials Measurement Laboratory, National Institute of Standards and Technology, Biosystems and Biomaterials Division, Gaithersburg, MD 20899, USA; vytautas.reipa@nist.gov

**Keywords:** DNA damage, standards, reference materials, mammalian cells, genotoxicity

## Abstract

As the potential applications of DNA diagnostics continue to expand, there is a need for improved methods and standards for DNA analysis. This report describes several methods that could be considered for the production of reference materials for the quantitative measurement of DNA damage in mammalian cells. With the focus on DNA strand breaks, potentially useful methods for assessing DNA damage in mammalian cells are reviewed. The advantages and limitations of each method, as well as additional concerns with respect to reference material development, are also discussed. In conclusion, we outline strategies for developing candidate DNA damage reference materials that could be adopted by research laboratories in a wide variety of applications.

## 1. Introduction

Exposure of mammalian cells to exogenous and endogenous factors, such as ionizing radiation, genotoxic agents, and mitochondrial redox decoupling, results in a wide range of adverse biomolecular effects, including lipid, protein, and DNA damage [1,2]. DNA damage is implicated in the development of mutagenesis, carcinogenesis, and pathogenesis of numerous diseases, including Parkinson’s, Alzheimer’s, and other severe disorders [3,4]. Accurate measurements of DNA damage are often critical in making diagnostic and therapeutic decisions for patients with these conditions [5]. In this regard, it is vital to have validated measurement methods and reference materials to quantify the extent of DNA damage and enable the comparison of data across various research and diagnostic labs.

As shown in Table 1, DNA diagnostics includes a wide range of applications, has an extensive global market, and has the potential for extensive use in DNA damage assays and standards. These applications include the fields of regenerative medicine, cancer diagnostics, drug testing, stress physiology, eco-toxicology, etc. [6]. Specific reference materials would be used in combination with commercial diagnostic kits as well as electrophoretic and chromatographic methods for measuring DNA damage (i.e., comet assay, H2AX immunoassay, mass spectrometry) [7,8]. These materials would also provide a resource for laboratories that use different measurement methods to compare their results and enable more robust interpretation.

Ionizing radiation, chemical agents, UV radiation [25], and a combination of these exposures lead to different types and amounts of DNA damage (**see** Table 2). This includes changes or lesions in the structure of the individual DNA bases and specific abasic sites. In addition to molecular damage, covalent binding of DNA to proteins can also occur, leading to the disruption of normal cell function [26]. A major type of damage can occur in the form of strand breaks, either in one strand or within both strands. Double-strand breaks are the most threatening to the organism and are the most challenging to repair by endogenous repair circuits [27].

Numerous assays exist for the analysis of damage to genomic DNA [28]. As shown in Table 2, these include the measurement of DNA strand breaks in whole cells using single-cell gel electrophoresis (comet assay) [29,30], a micronucleus assay [31], or the measurement of fragmented DNA in isolated DNA using capillary or gel electrophoresis [32,33]. The TUNEL assay, apoptosis analysis, 53BPI foci expression, as well as gene expression analysis, are also used in this regard [34,35,36,37]. In addition, oxidatively modified DNA lesions in isolated DNA can be measured using gas and liquid chromatography platforms (GC or LC) in combination with either electrochemical (EC) or mass spectrometry (MS or MS/MS) detection [38]. Measurement issues are often reported with these methods, resulting in a lack of agreement between laboratories, which is often quite significant [39,40,41]. For example, “Approximate agreement exists between different laboratories on human urinary levels of 8-OH dG; however, agreement on the levels of 8-OH dG in isolated DNA is very poor—values vary over 3 orders of magnitude” [3,42]. As a result, projects such as the European Standards Committee on Oxidative DNA damage (ESCODD) were set up to address problems associated with the measurement variability in the background levels of oxidative DNA damage [43,44].

DNA damage measurement methods currently available have varying sensitivities to different types of structural changes. For this reason, it is important to develop calibrants that match the individual assay method and the category of damage being evaluated [45]. Standard reference materials previously were available from the US National Institute of Standards and Technology (NIST) for mass spectrometry measurements of oxidatively induced DNA base lesions [46]. However, the currently available commercially produced methods and reference materials for measuring other forms of nucleic acid damage are inadequate to fully address the scope of this issue. These methods and materials are designed for specific diagnostic kits that are provided by commercial vendors. Universally adopted procedures and reference materials for the measurement of DNA damage in mammalian cells are highly desirable but currently unavailable. In addition, the use of specific positive controls is essential in demonstrating the extent to which a particular measurement method is performing as expected [47]. Some of the available prototype methods that have the potential to advance the development of these reference materials are briefly reviewed below.

Zainol et al. published a calibration device for a comet assay, where the DNA of the reference cells was substituted with bromodeoxyuridine (BrdU) [48]. By using a fluorescent anti-BrdU antibody, it was possible to differentiate the reference cells from the test cells when combined in the same gel used in the comet assay. This resulted in the reference cells being a genuine internal standard, thus reducing the measurement covariance compared to using separate gels for the test and reference materials. Although this method has the advantage of allowing the comparison of reference and test materials under identical conditions, it requires the additional quality control of the anti-BrdU antibody. In this regard, Brunborg et al. proposed a method where sample cells are mixed with reference cells from a distinct organism with a different genome size that have been irradiated and can be distinguished as comets of differing sizes [49].

Atha et al. proposed an alternative method of preparing cellular reference materials for comet assays using electrochemical DNA oxidation [50]. This method produced a linear increase in the percentage (DNA in the tail) of strand breaks under well-controlled oxidative environments and could be considered for making cellular reference materials for comet assays. This method would have the advantage of better reproducibility in producing multiple batches of reference materials due to well-defined oxidation conditions. otential prototype reference materials for comet assays that are produced by the chemical treatment of suspended cells by etoposide are also commercially available (BioTechne Inc.). Potassium bromate has been also tested as a positive control for Fpg-modified comet assays, demonstrating a concentration dependent response in cryopreserved samples [51]. These materials would benefit from further validation using additional methods to assess their fit-for-purpose usage. In a recent publication, Atha et al. examined three different chemical agents (etoposide, bleomycin, and ethyl methanesulfonate) that are known to have different mechanisms of action in producing DNA damage in mammalian cells [52]. This study illustrated the importance of using reference materials to establish method sensitivity across different measurement platforms.

The alkaline comet assay detects both single- and double-strand breaks. However, single- and double-strand breaks produce different electrophoretic patterns in the resulting imaged comet profiles. This leads to inaccuracies when the comet assay does not match the specific type of exposure and damage being measured. Using the comet assay as an example, it is paramount to use standard methods and specifically developed standards that match the type of exposure and DNA damage being measured [53,54].

In the following section, we present several methods we have utilized to prepare prototype samples that could serve as reference materials for DNA damage in mammalian cells, along with the assays we have used to assess the damage. These methods, as described in detail in our published work, have the advantage that they do not involve testing on humans or animals. Instead, they use commercially available cell lines that have been approved for experimental research. Since DNA strand breaks, particularly double-strand breaks, are difficult for the organism to repair, we highlight this type of DNA damage in our report of methods to be considered for the development of reference materials.

## 2. Methods to Prepare and Analyze Reference Materials

### 2.1. Preparation of DNA Damage Reference Materials

#### 2.1.1. Cell Type

##### Cultured CHO Cells

Chinese hamster ovary (CHO) cells are an ideal source of mammalian cells for the preparation of DNA damage standards. They are widely used and relatively easy to culture, harvest, and maintain. FreeStyle (CHO-S), otherwise known as suspension cell culture, provides a continuous stirring and uniform exposure of the cells to soluble chemical agents or nanoparticles that can otherwise settle and expose the cells as singles or small aggregates.

##### Other Mammalian Cell Types

Previously, we used cultured human neuronal blastoma (SH-SY5Y) and bronchial epithelial cells for our tests of manganese and CdSe nanoparticle toxicity. These cells were chosen as specific models for the human brain and lung cells during exposure to manganese and CdSe quantum dot nanoparticles [34,35,36]. Other human cell types such as HepG2 could be considered for other diagnostic applications [55].

#### 2.1.2. Chemical Treatment

##### Manganese and Potassium Bromide

Neuronal (SH-SY5Y) cells were grown in the presence and absence of (0 to 1000) µmol/L Mn^2+^ (MnCl_2_). This treatment resulted in extensive DNA base lesions, strand breaks, and a loss of cell viability [34]. Exposure to Mn^2+^ was used here to produce positive controls to assess manganese genotoxicity in neuronal cells using different assay systems. We do not have enough information to suggest that the use of this chemical is optimal for producing reference materials for DNA damage in other cell types. Alternatively, potassium bromate (KBrO_3_ ) is increasingly used to induce oxidative damage and may be more generally useful for producing reference materials [51].

##### Nanoparticles—CdSe Quantum Dots, Gold Nanoparticles

Bronchial epithelial (NHBE) cells were grown in the presence and absence of CdSe quantum dots for 24 h [35,36]. This method resulted in substantial DNA strand breaks by comet assay and an increase in micronuclei and 53BPI foci.

Human HepG2 epithelial cells were grown in the presence and absence of NIST gold nanoparticles for 24 h [55]. This resulted in the absence of DNA base lesions, as measured by mass spectrometry, and the conclusion that NIST gold nanoparticles do not induce oxidative DNA damage and could potentially serve as a negative nanomaterial-based genotoxicity control.

##### Chemical Genotoxic Agents

Etoposide is a podophyllotoxin glycoside with a D-glucose derivative that forms a ternary complex with DNA and topoisomerase II, prevents re-ligation of the DNA strands, and causes DNA strands to break. Cancer cells are predominantly affected due to their faster division. Treatment with this agent results in errors in DNA synthesis, which eventually induces apoptosis of the cancer cells. Chinese hamster ovary freestyle suspension (CHO-S) cells were exposed to (0 to 6) µg/mL etoposide for 1 h [52].

Bleomycin sulfate is a glycopeptide antitumor antibiotic. It induces DNA strand breaks, which, in vitro, depends on oxygen and metal ion concentrations. Its mechanism of action is not completely understood but it is thought that bleomycin chelates metal ions (primarily iron) and forms a pseudo-enzyme that reacts with oxygen to produce superoxide and hydroxyl radicals, which can induce base lesions and strand breaks. Freestyle suspension CHO-S cells were exposed to (0 to 2) µg/mL bleomycin for 1 h [52].

Ethyl methanesulfonate (EMS) is a potentially carcinogenic compound that causes point mutations in DNA through nucleotide substitution. The ethyl group of EMS reacts with guanine bases through alkylation, resulting in the formation of *O*^6^-ethylguanine. DNA polymerases then substitute thymine in place of cytosine opposite the *O*^6^-ethylguanine. As a result, during replication, the original G:C base pair becomes an A:T mutation. Freestyle suspension CHO-S cells were exposed to (0 to 1.6) mg/mL etoposide for 1 h [52].

#### 2.1.3. Electrochemical Oxidation

Oxidative stress is widely studied in biomedical toxicology [56]. Experiments designed to simulate an oxidative environment typically involve incubations with a particular concentration of a chemical oxidizer. However, such experiments with hydrogen peroxide are difficult to precisely repeat due to its high reactivity and subsequent instability. In these incubations, hydrogen peroxide (H_2_O_2_) serves as a cell oxidizer with a redox potential E_0_ = 0.88 V in an alkaline medium and can also serve as a possible source of hydroxyl radicals (●OH) in the presence of the transition metal ions, which act as catalysts in Fenton processes. Given the short lifetime (≈1 µs) and high reactivity (E_0_ = 2.8 V), of the hydroxyl radicals it is not possible to control the extent of the oxidation and relate damaged DNA products to the strength of the oxidative environment. This inevitably causes high variability and thwarts the comparison of results from different labs. Boron-doped diamond (BDD) electrodes have a high overvoltage for the electrolytic oxygen evolution via water oxidation and can directly generate reactive oxygen species (ROS) at high (E > 2 V) electrode potentials. This allows us to separate the direct oxidation conditions from the ROS-mediated action. By using electrochemical oxidation with BDD electrodes, we can induce DNA damage in cultured cells under controlled and reproducible redox conditions [50,57].

#### 2.1.4. Gamma Irradiation Treatment

Gamma irradiation is a widely used method to induce DNA damage. As an example, when exposed to radiation (0.2 Gy to 5 Gy), peripheral blood lymphocytes were shown to exhibit DNA double-strand breaks when monitored by a γ-H2AX assay [58]. Cassano et al. [59] used this method to produce a set of A549 cells with strand breaks and evaluated the variations in the comet assay data. Gamma irradiation can also be used to calibrate a comet assay to estimate the average number of DNA strand breaks per cell. This calibration is performed by treating the cells with ionizing radiation and measuring how this affects the comet % DNA in the tail [60]. This method utilizes a previous measurement through alkaline sucrose sedimentation showing that 1 Gy gamma radiation produces 0.31 breaks per 10^9^ Da of cellular DNA, or about 1000 breaks per diploid mammalian cell [61]. Our preliminary comet measurements of CHO-S cells after exposure to Cobalt-60 gamma irradiation of (2 to 4) Gy resulted in a % DNA in the tail of 20% to 25% or about 1000 to 2000 DNA breaks per haploid cell (unpublished data). Similar results were obtained with human lymphocytes in this range of exposure [60].

#### 2.1.5. UV Treatment

A practical form of radiation treatment is the exposure of cultured mammalian cells to near-ultra-violet light (UVA, 315 nm < l < 400 nm) and visible light [62] in the presence of photosensitizers [63] at doses ranging from 0 to 5 J/cm^2^. Visible spectrum photosensitizers such as Ro 19 8022 are widely used for base oxidation; however, their efficiency in producing strand brakes is limited [64]. To produce measurable DNA double-strand breaks that are detectable by a γ- H2AX assay, CHO cells require pre-exposure with the catalyst benzo [a] pyrene (BaP) at concentrations ranging from 10^−9^ mol/L to 10^−7^ mol/L [65]. Although BaP may interfere with some assay systems, it can be used as a tool to compare treatment conditions (presence of antioxidants, etc.) over a convenient period of time. Using the comet assay, we observed substantial DNA strand breaks at BaP concentrations above 10^−7^ mol/L [66].

#### 2.1.6. Viability Check

Viable cell numbers are assessed in triplicate after each chemical treatment using a cell counter, which is described in detail in [52]. Cell viability is measured after UV treatment using the MTS assay, which is described in detail in [66]. Other commercially available cell viability assays [67] can be employed but should be normalized against positive (CisPt or CdSO_4_) and negative (media) controls.

#### 2.1.7. Storage

The cells are typically spun down, the supernatant is discarded, and the cells are resuspended in a freezing medium (CHO expression medium + 10% DMSO). Then, 1 mL aliquots at a fixed cell density in the range of 10^6^ cells/mL are prepared, frozen using established techniques, and stored at −150 °C. For more details see [52].

### 2.2. Methods of DNA Damage Analysis

#### 2.2.1. DNA Base Lesions

##### Measurement of Modified DNA Bases by Mass Spectrometry

Samples containing genomic DNA are extracted from treated cells, enzymatically hydrolyzed, derivatized by trimethylsilylation, and analyzed using DNA extraction and isotope dilution GC–MS/MS and LC–MS/MS methodologies, as described in previous studies [38,55,68,69,70,71]. This method has the advantage of determining the exact structural changes in the DNA bases and quantifying them on a percentage basis. Although these methods are sensitive at the femtomole level to detect DNA base modifications, other methods have been shown to have a higher sensitivity to the detection of 8-oxodG [44]. Although the measurement of base lesions is not directly applicable to DNA strand breaks, it can provide important information to compare

#### 2.2.2. DNA Strand Breaks

##### Single Cell Gel Electrophoresis—Comet Assay

DNA single- and double-strand breaks, as well as alkali-labile sites, can be measured by a comet assay, as described previously [50,52,57,72]. This method is based on the migration of cleaved DNA out of the nuclei in an electric field, with the intact DNA remaining within the nucleoid. Imaging the comet’s tail and nucleoid allows for a relative assessment of the percentage of damaged DNA. This popular method has the advantage of directly examining the proportion of DNA resulting from strand breakage in individual cells [73,74]. Although widely used, the comet method can be subject to substantial variability and is difficult to quantify and automate, particularly without standard protocols and reference materials. However, it is relatively simple and efficient and can be performed using equipment commonly found in biological and clinical research labs [75]. Inter-laboratory validation studies have been performed using control materials produced by ionizing radiation [76]. Although comet assay control materials are available, they have not been universally adopted as reference materials. Some steps have been taken to standardize the comet assay protocol, including guidelines from experts at the International Workshop on Genotoxicology Test Procedures, IWGTP in 1999; the Organization for Economic Cooperation and Development, (OECD) [77]; and the International Comet Assay Workshop ComNet in 2011, which launched the comet network [78]. Guidelines have also been published with recommended criteria for performing comet assays to determine DNA single-strand breaks in eukaryotic cells [79,80].

In addition to DNA strand breaks, the comet assay has been adapted to detect other types of DNA damage, including damaged bases, apurinic and apyrimidinic sites, bulky adducts, and inter- and intrastrand crosslinks (reviewed by Cordelli et al. [81]). These modifications have made the comet assay a more versatile tool in the testing of a wide range of genotoxic agents [81].

Modifications of the comet assay, such as the combination of fluorescence in situ hybridization (FISH) and the comet assay (comet FISH), have been developed to detect sequence-specific damage [82,83]. Additionally, the addition of lesion-specific repair enzymes formamidopyrimidine DNA glycosylase (FPG), 8-hydroxyguanine DNA glycosylase (hOGGI), and endonuclease III (ENDO III) has increased the sensitivity of the comet assay to detect base lesions [84]. The presence of DNA crosslinks can be detected by comparing test cells treated with a crosslinking-inducing agent or extreme electrophoresis conditions followed by ionizing radiation to untreated cells [85]. Similarly, bulky DNA adducts can be detected by comparing cells treated in culture with inhibitors of the nucleotide excision repair pathway to untreated cells [86]. Although these procedures are more complicated due to the inclusion of additional steps in the comet assay and must be carefully interpreted using the proper controls, they increase the versatility of the comet assay [81].

The Comet-Chip procedure has also been developed, which allows a more even distribution of cells to be imaged in the comet assay and helps streamline the process [87]. This variation can be an advantage but requires an additional filtering step and it is yet to be fully adopted by the user community. In this context, we have developed an automated imaging system to aid in the process of gathering the required data for the proper statistical analysis of comet slides [72]. In addition, there have been more recent publications, which describe the steps required to standardize comet assay procedures [88,89].

##### Gamma H2AX Assay

A γ-H2AX assay is commonly used to assess DNA double-strand breaks in human cultured cells, peripheral blood mononuclear cells, and tissue biopsies. This immunohistochemical method has also been adapted for ELISA and flow cytometry platforms, which have the advantage of automation and high throughput [90]. However, the assay is indirect and relies on proportional chemical response signals in the affected cells. These signals can be affected by the condition of the cells and their viability after treatment [91]. The measurement of γ-H2AX-phosphorylated histone protein is subject not only to variations in the antibody and labeling used for detection but also to interference from cellular proteins. Repeatability measurements should also be taken before and after the storage of the cells.

## 3. Discussion

DNA damage reference materials could be used in a wide range of research and diagnostic areas that require an appraisal of oxidative DNA damage in mammalian cells. Some of these tests such as the comet assay require the use of viable mammalian cells. Reference materials in the form of viable mammalian cells could be used as a control for DNA-damaging artifacts that may occur during treatment and the isolation and purification of DNA. We observed that CHO-S cells treated with etoposide, bleomycin, and ethyl methanesulfonate each retained 90% viability at genotoxic concentrations, which produced a wide range of DNA damage that could be measured by a comet assay [52]. Ethyl methanesulfonate exposure for 4 h at 1.6 mg/mL was particularly effective in producing DNA strand breaks (80% DNA in the tail) with less than a 10% loss in cell viability. However, since bleomycin was the only treatment method that also produced measurable DNA lesions by mass spectrometry [52], this method would be useful in the design of a reference material that contains both DNA base lesions and strand breaks.

Although other methods can be used for the analysis of comets, such as the olive tail moment (OTM) [92], the % DNA in the tail was chosen in our previous study since it is appropriate for regulatory and interlaboratory comparison studies with minimal variability [93]. In addition, the % DNA in the tail is a well-defined parameter that describes the proportion of DNA resulting from strand breakage and can be directly compared to the % loss in cell viability. In studies where differences in the type of DNA damage (i.e., crosslinks, single- and double-strand breaks) need to be detected, the use of the olive tail moments may be preferable, as it depends on the shape of the comet tail and is sensitive to the types of damaged DNA [92,93]. In this regard, the certification of a reference material using multiple methods, such as the % DNA in the tail and OTM, would be useful.

In the development of standard reference materials for DNA damage, it will be necessary to characterize these materials to include the specific analysis method that will be used in a particular application. Cytology-based assays, such as micronucleus, TUNEL, and 53BPI foci assays, may be especially difficult to standardize in this regard due to the various optical instruments and reagents available. However, for a reference material specifically designed to standardize the measurement of DNA strand breaks by a comet assay, it is still helpful to validate it with complementary cytology-based assays. Additionally, it would be useful to characterize such a material with an orthogonal technique such as capillary electrophoresis [32]. An essential but challenging parameter to include in the characterization of a cellular reference material is the determination of the average number of DNA strand breaks per cell. As mentioned earlier for treatment by gamma irradiation, this can then be used as a calibration for the comet assay and other assays of biological importance [60].

The reference materials must be stable during storage and shipment to the user and in sufficient supply to distribute to the various laboratories participating in a coordinated study. Important cryopreservation procedures have recently been described [16,94]. One particular concern with chemical methods used to produce DNA damage is that residual amounts of the genotoxic agent may continue to cause further damage, even after characterization and during storage. Although this effect has not been thoroughly documented, it may be an issue with biochemical assay systems such as the γ-H2AX assay, which relies on functioning enzymes for proportional signaling of the double-strand breaks. For this reason, treatments such as g-ray or UV exposure may produce less interference with these enzyme systems.

In order to produce multiple batches of materials with consistent levels of DNA damage, the genotoxic treatment exposure needs to be precisely controlled (timing and intensity). Electrochemical and ionizing radiation treatments allow such control. UV exposure using available and inexpensive UV sources can also be used to control exposure without the costly and problematic safety-related issues associated with g-ray sources.

An additional concern with cellular reference materials is that the repair enzymes normally present in the cell are still active before the damage assay can be completed. One possible way to resolve this would be to flash freeze and store the reference materials at −150 °C, followed by a rapid assay after thawing, thus slowing down and reducing the repair process. In addition, double-strand breaks are the most difficult type of DNA damage for the cell to repair and they often require more time to be repaired than other types of DNA damage. As a result, this type of DNA damage should be more stable than others, particularly when stored at −150 °C.

A single preparation of mammalian cells consisting of a determined (certified) proportion of each type of DNA damage (i.e., alkylated and oxidized bases, abasic sites, crosslinks, and strand breaks) would be ideal as a candidate reference material. However, this would be difficult to produce and characterize separately by several different methods. Although it is preferable to match the treatment used to produce the reference material to the sample being tested, a reference material for DNA single- and double-strand breaks could be efficiently produced and characterized, as described in this report, using cultured suspension cells treated chemically with EMS or UV/BaP and characterized by a comet assay [52,66]. Since different cell types may respond differently to the various reagents used in the comet assay system (differences in membrane permeability, chromosomal structure, etc.), it is preferable to use the same cell type for both the reference material and the assay system. Haploid mammalian cells such as CHOs may be preferable for fertility measurements, whereas diploid cells such as Jurkat suspension cells may be preferable for other diagnostic measurements. The characterization of this material could then be supplemented with additional methods such as a γ-H2AX assay, which specifically detects double-strand breaks in live cells. This could also broaden the applicability of the reference material, allowing it to be used as a control for a γ-H2AX assay. Micronucleus, TUNEL, and 53BPI foci expression assays may also be useful for assessing the biological effects of DNA damage but would require standardization. In addition, viability assays could be useful as a gauge of the overall biologic impact of DNA damage.

## 4. Conclusions

With the increasing number of DNA diagnostic applications, the potential applications for DNA damage measurements are also expanding. This will result in an increasing demand for improved measurement methods and reference materials to ensure the accuracy of clinically important measurements and the quality of manufactured DNA products. Although many improved methods have been proposed, including those we have utilized, this report provides a comprehensive overview of potential methods for both the production and characterization of DNA damage reference materials, addressing the current and future needs of the DNA diagnostics community.

**Note:** Certain commercial equipment, instruments, and materials are identified in this paper to specify an experimental procedure as completely as possible. In no case does the identification of particular equipment or materials imply a recommendation or endorsement by the National Institute of Standards and Technology nor does it imply that the materials, instruments, or equipment are necessarily the best available for the purpose.

## Figures and Tables

**Table 1:** DNA diagnostics/potential stakeholders in DNA damage testing.

DNA Diagnostics Categories *	DNA Key Players/Potential Stakeholders **	DNA Global Market Analysis **	DNA Damage Refs ***
DNA Diagnostics (Major):			
Genetic Testing	BioRad Labs, Abbot Labs, Myriad Genetics, Danaher, Hoffmann-LaRoche, Illumina, Eurofins, Qiagen, ThermoFisher, CSI, Ltd.	12.7 B USD in 2019, 21.26 B USD by 2027, 10 % CAGR (2019–2027).	[9,10]
Next-Generation Sequencing	Illumina, ThemoFisher, Pacific Biosystems, Beijing Genomics, Qiagen, Agilent, Bio-Rad, PerkinElmer, Genomatix, Oxford Nanopore, New England Biolabs, Myriad Genetics.	4.5 B USD in 2018, 18.6 B USD by 2026, 19.2% CAGR (2019–2026)	[11]
Cytogenetic Screening	Abbot Labs, Agilent, Irvine Scientific, Applied Spectral Imaging, Bio-Rad, PerkinElmer, Sysmex, ThermoFisher, OPKO health, Hoffman-LaRoche, Metasystems	1.5 B USD in 2017, 3.2 B USD by 2025, 9.5% CAGR (2018–2025).	[12]
Gene Therapy	Adaptimmune, Anchiano, Achieve, Adverum, Abeona, Applied Genetic Technol., Arbutus, Merk, Celgene, Shanghai Sunway, BioCancell, Shenzhen SiBiono, SynerGene, OxoGenex Pharmaceuticals, Novartus, Amgen, Genetech	393 M USD in 2018, 6.2 B USD by 2026, 34% CAGR (2019–2026).	[13]
Cancer Gene Therapy	Adaptimmune, GlaxoSmithKline, BluBird Bio, Merk, Celgene, Shanghai Sunway, BioCancell, Shenzhen SiBiono, GeneTech, SynerGene, OxoGenex Pharmaceuticals	289 M USD in 2016, 2.1 B USD by 2023, 32.4% CAGR (2020–2027).	[14]
Epigenetics	Abcam, ActiveMotif, Agilent, Merk Diagenode, Millipore, Illumina, PerkinElmer, ThermoFisher, Zymo Research, Qiagen	772 M USD in 2019, 2.2 B USD by 2027, 13.6% CAGR (2020–2027).	[15,16]
DNA Diagnostics (Minor):			
Pre-Implantation Screening	Illumina, ThermoFisher, Agilent, PerkinElmer, Coopersurgical, Abbot Labs, Natera, Oxford Gene Technol., Yikon Genomics, SiGene		
Blood Bank (Cytogenetic Screening)			[17,18,19]
Gene modification (CRISPR, DNA production artifacts/ stability testing)			[20]
Male Fertility Testing			[21]
Animal Genetics	Genus, Topigs Norsvin, Envigo, CRV Holding, Hendrix Genetics, Neogen Animal Genetics, Alta Genetics, Zoetis		
Environmental (EPA) (Genotoxicology)			[22,23]
Forensics (NIJ) (Crime Lab Identification) (DNA artifacts/stability)			[9]
Ancestry, Archeogenetics (DNA artifacts/stability)			

* CRISPR, clustered regularly interspaced short palindromic repeats; EPA, Environmental Protection Agency; NIJ, National Institute of Justice. ** DNA Diagnostics Key Players and Market Analysis by Allied Market Research, Allied Analytics, LLP [24]. The list of key players/potential stakeholders and the global market analysis obtained from Allied Market Research are continually evolving and is an indication of commercial activity at the time of publication. It should not be considered a complete list or reference for investment. USD, US dollars; CAGR, Compound Annual Growth Rate. *** Specific examples where the accurate measurement of DNA damage plays an important diagnostic role.

**Table 2:** DNA damage in mammalian cells/types of damage and how it is measured.

Types of DNA Damage	How Damage is Produced *	How Damage is Measured **	Commercial Kits ***
Base Modification/Lesions:			
			
Alkylated bases:	Alkylating agents, UV (C)	Mutational Profile	
(O^6^ methyl guanine)		Sequencing, LC-MS	Genetic Signatures
(N^7^ methyl guanine)		Electrochemical	(Methyl Easy)
Oxidized bases:	Reactive oxygen species	HPLC-ED, LC-MS/MS	EpiGentek Inc. (Epiquik)
(8-OH guanine)		ELISA assay	Cell Biolabs Inc. (OxiSelect)
			
Abasic Sites:	Reactive oxygen species	Sequencing	American Research Products
Apurinic	Hydrolysis	ELISA assay	(ARPELISA)
Apyrimidinic			Abcam, Inc.
			
Crosslinks:	UV (C), PAH, BAP		
Interstrand	Aflatoxin	LC-MS/MS	Arbor Assays, Inc.
Intrastrand		ELISA assay	Abcam, Inc.
DNA–Protein			
			
Strand Breaks:	Reactive oxygen species	Electrophoresis	R&D Systems, Inc.
Single	Hydrolysis	HPLC	Metasystems, Inc.
	Ionizing radiation, X-rays	Comet assay	(CometScan)
			
			
Double	Ionizing radiation, Xrays	Chromosomal Aberation	Metasystems, Inc.
	Genotoxic agents	Micronucleus assay	MetaCyte) (MNScore)
	Anti-tumor drugs	Comet assay γ H2AX assay	Bio-Techne, Inc.
			(MNScore)

* UV (C), ultra-violet light (200–280 nm); PAH, polycyclic aromatic hydrocarbon; BAP, benzo [a] pyrene. ** LC-MS, liquid chromatography–mass spectrometry; HPLC-ED, high-performance liquid chromatography–electrochemical detection; ELISA, enzyme-linked immunosorbent assay; comet assay, micronucleus assay, and H2AX assay (see Section 2). *** Genetic Signatures, Newtown, Australia; Epigentek Group Inc., Farmingdale, NY, USA; Cell Biolabs Inc., San Diego, CA USA; American Research Products, Waltham, MA, USA; Abcam, Inc., Waltham, MA, USA; Arbor Assays Inc., Ann Arbor, MI USA; R&D Systems Inc., Minneapolis, MN USA; Metasystems, Altlussheim, Germany; Bio-Techne Corp., Minneapolis, MN USA.. Commercial calibration kits are provided as examples and are not a complete list of available kits.

## Data Availability

The data presented in this review is available in published form.
